# CRISPR/Cas-Mediated Genome Engineering in Plants: Application and Prospectives

**DOI:** 10.3390/plants13141884

**Published:** 2024-07-09

**Authors:** Swetaleena Mishra, Subhendu Nayak, Narendra Tuteja, Sowmya Poosapati, Durga Madhab Swain, Ranjan Kumar Sahoo

**Affiliations:** 1 Department of Biotechnology, Centurion University of Technology and Management, Bhubaneswar 752050, India; 220506292002@cutm.ac.in; 2 Vidya USA Corporation, Otis Stone Hunter Road, Bunnell, FL 32100, USA; subhendu@vidyaherbsusa.com; 3 Plant Molecular Biology Group, International Centre for Genetic Engineering and Biotechnology (ICGEB), New Delhi 110067, India; narendratuteja@gmail.com; 4 Plant Biology Laboratory, Salk Institute for Biological Studies, San Diego, CA 92037, USA; 5 MU Bond Life Sciences Center, University of Missouri, Columbia, MO 65211, USA

**Keywords:** CRISPR-Cas system, crop improvement, genome engineering, prime editing

## Abstract

Genetic engineering has become an essential element in developing climate-resilient crops and environmentally sustainable solutions to respond to the increasing need for global food security. Genome editing using CRISPR/Cas [Clustered regulatory interspaced short palindromic repeat (CRISPR)-associated protein (Cas)] technology is being applied to a variety of organisms, including plants. This technique has become popular because of its high specificity, effectiveness, and low production cost. Therefore, this technology has the potential to revolutionize agriculture and contribute to global food security. Over the past few years, increasing efforts have been seen in its application in developing higher-yielding, nutrition-rich, disease-resistant, and stress-tolerant “crops”, fruits, and vegetables. Cas proteins such as Cas9, Cas12, Cas13, and Cas14, among others, have distinct architectures and have been used to create new genetic tools that improve features that are important for agriculture. The versatility of Cas has accelerated genomic analysis and facilitated the use of CRISPR/Cas to manipulate and alter nucleic acid sequences in cells of different organisms. This review provides the evolution of CRISPR technology exploring its mechanisms and contrasting it with traditional breeding and transgenic approaches to improve different aspects of stress tolerance. We have also discussed the CRISPR/Cas system and explored three Cas proteins that are currently known to exist: Cas12, Cas13, and Cas14 and their potential to generate foreign-DNA-free or non-transgenic crops that could be easily regulated for commercialization in most countries.

## 1. Introduction

The world population is about to reach 10 billion in the coming years. This increased rate of population rise would result in increased global need for food followed by production [1]. However, the reduction in agricultural land caused by rapid urbanization and industrialization and reduction in fresh produce due to extreme weather accompanied by environmental stressors like climate change and global warming limit agriculture and food production, which may threaten food security [2]. Breeding practices have been helpful over decades in providing us improved crops. However, one of the barriers to de novo domestication through gene editing is the lack of expertise of botanists in wild-plant biology, and, in recent times, we are in need of novel and efficient tools that could improve crop traits in a shorter time frame. Various techniques have been developed that allow us to modify gene sequences specifically, such as transcription activator-like effector nucleases (TALENs), zinc finger nucleases (ZFNs), and CRISPR/Cas9, that have revolutionized crop engineering by making it easier to create crops with higher yield and other desirable characteristics. Among them, the best state-of-the-art technology is CRISPR/Cas genome editing, which is gaining popularity because of its border application across different organisms [3]. Different genome editing methods have been well compared and the potential use of CRISPR/Cas9 technology in precision plant breeding has been reviewed in detail by Sun et al., 2016 and Chen et al., 2019 [4,5]. In recent years, there has been a significant growth in the quantity and variety of known CRISPR-Cas systems [6]. CRISPR–Cas system actually originated from the defence mechanism that bacteria use based on RNA, and they recognize and eliminate foreign DNA from invading plasmids and bacteriophages and are thus considered a type of bacterial “immune system” [7]. Overall, the CRISPR/Cas9 modifies genes through cutting of DNA, followed by the natural mechanisms of DNA repair [3,8]. The adoption of genome-edited plants by the general public and regulatory agencies is also impacted by these strategies [9].

In this review, we provide an overview of the most recent developments in CRISPR/Cas technology, the functional processes of all identified Cas proteins, and their use in contemporary horticulture and agriculture.

## 2. History of CRISPR/Cas System

**Figure 1:**
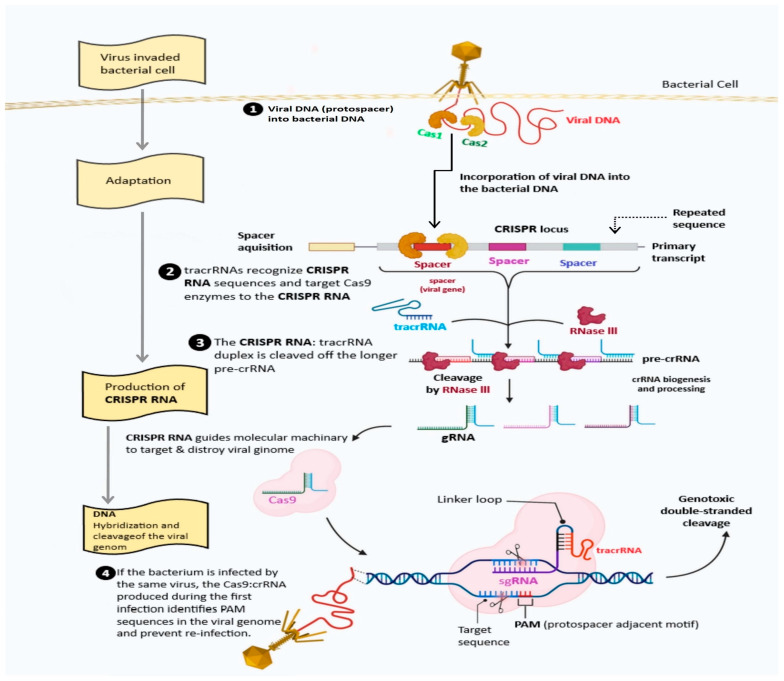
The CRISPR/Cas adaptive immunity system involves three stages: CRISPR adaptation, CRISPR RNA biogenesis, and CRISPR interference. In the adaptation stage, viruses trigger Cas1 and Cas2 modules, cleaving invading sequences. In the biogenesis stage, the CRISPR array is transcribed into mature crRNA molecules, forming effector complexes with Cas proteins.

Clustered Regularly Interspaced Short Palindromic Repeats (CRISPR) was first identified in *Escherichia coli* in 1987 by a Japanese scientist, Yoshizumi Ishino, and his team, as unique repetitive DNA sequences interspersed with spacer sequences [10]. Later, such sequences have been identified in Salmonella Enterica and Shigella dysenteries, two types of enterobacteria, as well as in other *E. coli* strains. Similar to this, when examining Mycobacterium TB strains, researchers found 36 bp repetitions scattered with unique 35–41 bp spacers. In the ensuing investigations, this CRISPR array was found in the genomes of 40% of archaea and 90% of bacteria. The function of CRISPR was a mystery for some time due to a lack of required spacer or protospacer genome sequence data. It is only in the early 2000s that it was discovered that the bacteria possessing these homologous spacer sequences present in bacteriophages and viruses were immune to the attack, suggesting their role in adaptive immunity in prokaryotes. Following this research, it has been found that when a bacterium is challenged by a virus, spacer sequences in CRISPR array are transcribed and guided by Cas protein to cleave the viral DNA or RNA to prevent further infection. In addition to the requirement of Cas protein, the CRISPR system also needs a short 2–6 nucleotide protospacer adjacent motif sequence (PAM) placed adjacent to the sequence identified by the Cas protein (Figure 1). This ability of the bacterial adaptive immune system and the ease of selecting the target genomic location based on shorter PAM requirements has resulted in precise genome editing in various organisms for varied applications [11].

## 3. Nomenclature and Mechanism of CRISPR/Cas System

The CRISPR-Cas system has been divided into two main categories and several subtypes based on the complexity of the effector proteins, genomic location, pre-crRNA processing and interference. The diversity of CRISPR-Cas has increased over the past years and the evolutionary classification of this system and Cas genes has been well documented by Makarova et al., 2019 [6]. In this review, we have discussed the two main classes of CRISPR-Cas system with focus on Cas proteins that have the potential for use in agriculture.

The CRISPR-Cas system is mainly classified in two main classes, I and II. In the Class 1 system, multiple effector proteins are required for the RNA-guided target cleavage, while in Class 2, only one RNA-guided endonuclease is required for the DNA sequence cleavage. Three types of CRISPR are present in the Class 1 system: I, III, and IV, while three types are present in the Class 2 system: II, V, and VI. The Cas3 signature gene, which codes for an immune protein containing a helicase to unravel DNA–DNA and RNA–DNA duplexes, is found in the type I system’s CRISPR/Cas locus. The multidomain Cas9 (CRISPR-associated protein 9) that the type II locus generates cleaves and targets dsDNA. The cas10 signature gene is present in type III CRISPR/Cas, along with a multidomain protein with a palm domain that can target ssDNA. Other Cas proteins like csf1 (large subunit, cas8-like) signature protein belongs to type IV locus and the RuvC gene, encoded by the CRISPR from Prevotella and Francisella 1 (Cpf1), C2c1, or C2c3 protein, which are carried by the type V locus, which carries the Cas12 signature gene (a DNA repair-related *E. coli* protein) domain. This domain cleaves either dsDNA or ssDNA. Type VI has a nucleotide-binding domain (HEPN) called Cas13 (C2c2) that is responsible for cleaving ssRNA in higher eukaryotes and prokaryotes (Table 1).

Different groups of bacteria and many Archaea use different CRISPR systems to defend themselves against foreign nucleic acids, such as viruses and plasmids. The taxonomic distribution of the major classes of CRISPR-Cas systems in different groups of bacteria and Archaea have been well documented to show the distribution of different Cas proteins in these organisms [18]. The CRISPR-Cas system could be explained to act in three stages: adaptation, expression, and interference. Briefly, following the infection by the viruses, the bacterial host genome’s CRISPR repeat sequences are decoded as arrays, and type II CRISPR systems integrate sequences from overrunning DNA between them. The CRISPR reprise arrays are repeated to form crRNA, each containing a different sequence taken from the DNA it overlaps, called the “protospacer” sequence. This sequence makes up a part of the CRISPR reprise. In the type II system, the trans-activating crRNA (tracrRNA), an alternative RNA, hybridizes with each crRNA, forming a complex with the Cas9 nuclease. Only when PAMs are located conterminous to the protospacer-decoded portion of the crRNA will Cas9 be directed to stick reciprocal target-DNA sequences to make breaks in the DNA [19]. A detailed classification of these CRISPR-Cas systems and Cas proteins would provide valuable information for modifying and adapting them for various applications.

## 4. Cas Proteins of the CRISPR System

Cas proteins are an essential component of CRISPR systems and their varied abilities to edit DNA or RNA make them a potential tool in genome editing. Cas proteins, including Cas9, Cas12, Cas13, and Cas14, have been exploited for genetic engineering [7] and are discussed further below (Table 2 and Table 3).

### 4.1. Cas 1 and Cas 2 Proteins

The Cas1–Cas2 exonuclease system is ubiquitous in all the CRISPR-Cas systems, and it helps in cleaving both the target DNA and CRISPR array, a crucial step in CRISPR-Cas mechanism. Cas1 and Cas2 proteins are found in E. coli K12, forming a hexameric complex. Cas1 is an asymmetric homodimer with a central ferredoxin fold, while Cas2 is a symmetric homodimer with a central ferredoxin fold [19]. The complex produces crystal diffracting X-rays with a resolution of 2.3 Å. Both are heterozygous molecules that catalyse spacer integration via transesterification reactions. Recent studies have shown that Cas1–Cas2 proteins can recognize the PAM containing prespacer in the reannealing DNA in repair complexes, and could flexibly coordinate with other accessory proteins to process prespacers and directionality of integration. Cas1–Cas2 protects the host CRISPR system by cleaving the PAM after initial integration of the prespacer into the host CRISPR array and hence could potentially be used as an alternative to systems lacking Cas4, a well-studied protein that acts as a PAM-processing endonuclease. In spite of these advancements, it is still unclear regarding the upstream CRISPR substrate biogenesis, and future investigations into their precise role in adaptation of the CRISPR-Cas system would open avenues for their wide spread application in genome editing [45].

### 4.2. Cas9 Protein

**Figure 2:**
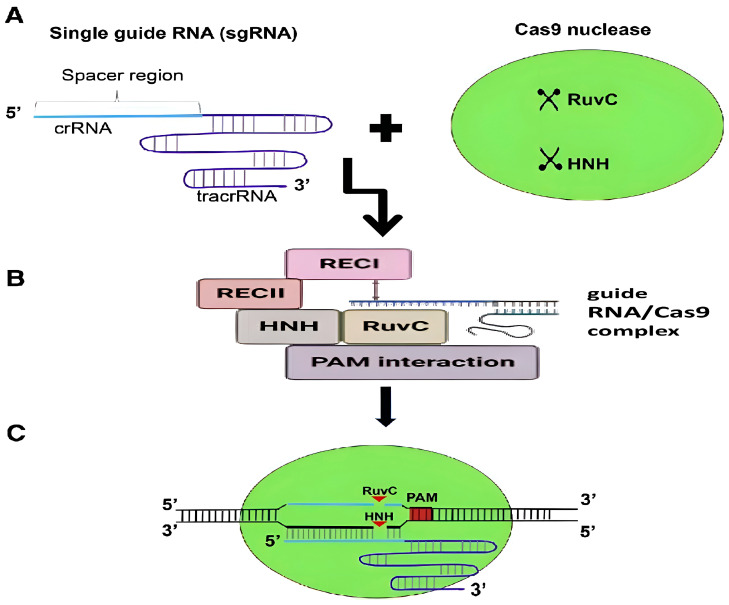
Illustration of CRISPR-Cas9 mechanism: (**A**) CRISPR array is transcribed into precursor crRNA, which are then cleaved into mature crRNA to form effector complexes with Cas9 protein (**B**) The CRISPR/Cas9 mechanism involves six domains: recognition flap (REC I), arginine-rich bridging helix, PAM interaction, HNH, and RuvC. REC I is responsible for hRNA binding, while HNH and RuvC initiate cleavage. B-programmed gRNA binds to Cas9, transforming it into an active form. (**C**) Cas9 generates a 3bp DSP using HNH and RuvC domains.

Cas9, formerly known as Csn1 or Csx12, is a protein connected to the Streptococcus pyogenes CRISPR mechanisms of adaptive immunity. Cas9 protein is the first Cas protein to be employed in genome editing (SpCas9) and its mechanism of action is extensively researched and applied in various organisms [46]. In natural and synthetic CRISPR/Cas systems, the SpCas9 protein functions as a DNA endonuclease and consists of 1368 amino acids in its big multifunctional domain. Cas9 endonuclease consists of six domains, of which two are nuclease domains, RuvC and HNH, and one is a PAM-interacting domain. The primary function of Cas9 protein is to cut three base pairs upstream of the PAM sequence in dsDNA creating double stranded breaks. Since the CRISPR/Cas9 system was created by fusing twin tracrRNA:crRNA into a single-guide RNA (sgRNA), it could cut specific target dsDNA or ssDNA sequences (Figure 2). The cuts generated via the CRISPR-Cas9 system could be repaired either by non-homologous end joining (NHEJ) repair or homology-directed repair (HDR). HDR usually requires a template that could be a sister chromatid or exogenously supplied DNA (gene knock-in) with homology arms to DSBs [47]. CRISPR-Cas9 is a preferred tool over other genome editing tools like ZFN and TALEN systems due to its simple design and higher effectiveness. The ability to create and use several sequence-specific gRNAs simultaneously allows for multiplex genome editing and makes it a prime trait development tool. Researchers have widely adopted this system in various fields, including microorganisms, plants, animals, insects, and human cell lines [48]. Despite numerous advances, CRISPR/Cas9 systems have numerous failings that raise numerous questions about the pitfalls involved in editing. Few major concerns of Cas9 application are on- and off-target mutations and restriction of edits to regions of high GC content because of a “G”-rich PAM sequence requirement (Bernabé-Orts et al., 2019, [49]) of this protein. Indeed, the sophisticated CRISPR/Cas systems with HDR produce unwanted mutations. Still, these off-target mutations can be eased by using a modified interpretation of Cas9 called Null-Cas9 (dCas9), which lacks endonuclease activity [50]. Cas9 could be used for epigenome editing rather than creating irreversible genome modifications. However, further studies are necessary to improve the specificity of Cas9 function and to reduce off-target mutations [51].

### 4.3. Cas12 Protein

The CRISPR-Cas system, which was partially repurposed as a programmable genome-editing tool, was identified as an adaptively susceptible mechanism in prokaryotes [52]. In programmable genome editing, the Cas9 and Cas12a proteins are widely utilised. In contrast to Cas9, Cas12a protein has the ability to recognize “T”-rich PAM sequences and generate staggered ends that could promote efficient site-directed integration in comparison [18]. As most of the regulatory elements are “AT” rich, Cas12a could serve as an efficient tool for engineering epigenome modification [53]. Cas12a consists of two main components, protein/effector nuclease and a single crRNA, which are sufficient to process the crRNA unlike Cas9 which requires a tracrRNA to form a mature crRNA [54]. Three homologs of Cas12a viz., FnCas12a (from *Francisella novicida*), LbCas12a (from *Lachnospiraceae bacterium*), and AsCas12a (from *Acidaminococcus* sp.) share similar domain architecture and are widely utilized in plant genome editing technologies [16]. In a recent report, two other homologs of Cas12a viz., Ev1Cas12a and Hs1Cas12a, have been shown to produce efficient multiplex genome editing in rice and tomato protoplasts [55]. Cas12a editing has been applied to various crops including rice, wheat, tomato, citrus, soybean, and the model plant Arabidopsis thaliana [56] with varied editing efficiencies. Recently, miniature variants, CRISPR/Cas12j (CasΦ) and Cas12f systems, were discovered with less than half the size of Cas9 and their orthologs were successfully tested in plants [29]. Efforts are underway to generate improved versions of Cas12a with altered PAM specificities [57,58] and improved temperature sensitivity for its flexible application and efficient delivery into plant systems [59].

### 4.4. Cas13 Protein

**Figure 3:**
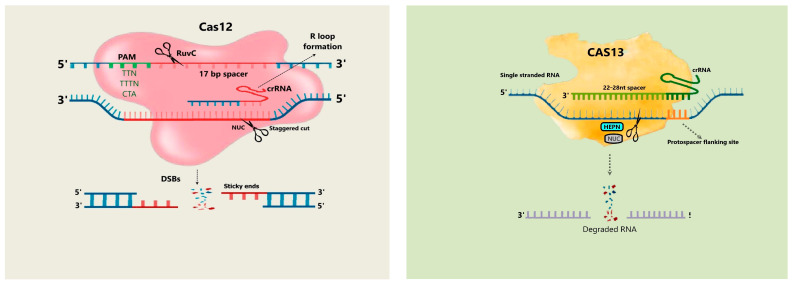

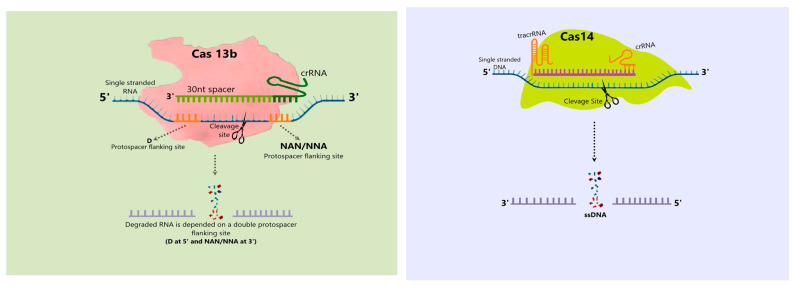
The CRISPR/Cas12 mechanism involves cleaving the target region near the PAM sequence, creating an R-loop and R cycle. The CRISPR/Cas13a mechanism activates Cas13a protein, which encodes crRNA, NUC particles, and HEPN domains for target RNA. The CRISPR/Cas14 system consists of Cas14 protein, tracrRNA and crRNA, Case 14, and target proteins of ssDNA. The PAM domain meets usability and application criteria for future genetic engineering.

Cas13 proteins are displayed in at least 21 bacterial genomes, and they consist of two distinct HEPN (higher eukaryote and prokaryote nucleotide binding) domains and a single protein effector [60] (Figure 3). CRISPR-Cas13 is the only known system among others to target single-stranded RNA, and, owing to this ability, Cas13 has a potent application in plants to target RNA (coding and non-coding) and silence defence responses of RNA viruses [61]. Recent studies have shown that Cas13 independent guide-induced gene silencing (GIGS) could substantially reduce the viral load in tobacco, tomato, and Arabidopsis, even in the absence of Cas13 proteins, offering a potential system for studying tissue- or time-specific expression studies which are otherwise difficult to manipulate with other CRISPR-Cas systems [62]. Cas13a orthologs LwaCas13a (Cas13a from *Leptotrichia wadei*) and LshCas13a (Cas13a from *Leptotrichia shahii*) were successfully tested in plants with moderate efficiency. The Cas13d subtype, which is a smaller protein than Cas13a, has been discovered to perform efficiently at a broad temperature range of 24–41 °C. Modified and programmable Cas13 mutant forms (dCas13 and Cas13x) were generated to effectively target specific effectors to specific RNAs in order to elicit specific modifications [63]. RNAs can be selectively targeted to use Cas13 entirely because of the innate crRNA biosynthesis. In contrast to RNA interference, genome changes induced by CRISPR/Cas13 are not restricted to focusing on cytoplasmic transcripts. In expansion, Cas13 selectively knocks off cytoplasmic mRNA transcripts, enabling quicker downregulation of expression [63]. These finding suggests a promising opportunity to deploy this system for generating multigene silencing in polypoid plants [60,64].

### 4.5. Cas12f Protein

Cas12f, a protein in the CRISPR-Cas framework, has been characterized for its unique biochemical properties. This protein originated in extreme thermophiles and shows high affinity to single-stranded DNA (ssDNA) without the requirement of a PAM sequence for its activity [27] (Figure 3). The CRISPR/Cas12f system has been used to detect and genotype single-nucleotide polymorphisms (SNPs) and improve resistance to ssDNA viruses in crops [65]. Cas12f protein offers several advantages over the traditional Cas9 protein. For example, Cas12f is incredibly small, with only 500 amino acids, and hence could be delivered to any target organism more efficiently than the Cas9 protein (Table 4 and Table 5). Moreover, Cas12f-based biosensors (HARRY) are receiving more focus than others like Cas12 and Cas13 proteins because of their improved sensitivity in the detection of diverse targets [66].

The major CRISPR-Cas systems which are currently in use have different pros and cons and there is always a scope to improve their efficiency and build up new approaches to use these systems for varied application across different fields.

## 5. Prime Editing

The double-stranded breaks (DSB) caused by the CRISPR-Cas9 system usually generate a complex mix of unintended in-del byproducts, translocations, and chromosome fragmentations. Although attempts have been made to improve these issues [69,70], they are not accurate enough, and with advancements in knowledge of the CRISPR-Cas mechanism, researchers are continuously working on improving and optimizing this gene editing technology. One such technology is named prime editing, developed by David Liu’s group in 2019. This technology has been shown to have reduced off-target effects because of its unique ability to search and replace target sequences without the need of an exogenous donor repair template [71,72]. The prime editing system mainly consists of two components: a prime editor (PE-nCas9-MMLV) and a PE guide RNA (pegRNA), and its mechanism of action is well studied, and improved versions of prime editors have been developed for better accuracy [73]. Because of this unique feature of the prime editing system, it has been successfully used to develop resistance against biotic stress by integrating a 30-nucleotide cis-regulatory element through knock-in with high efficiency in rice [74]. Various methods used to improve prime editing technology have been summarized by [73,75]. Improved versions of the PE system have been successfully used to generate crop germplasm resources, herbicide tolerance germplasms in rice and wheat by generating specific edits, and also designing mutations at existing variant sites [7,73,76]. By using a modified prime editor, pCXPE03 in tomato, three genes, viz. GAI, ALS2, and PDS1, were edited efficiently and produced a lower frequency of off-target byproducts of 0.5–4.9% [77]. In addition to these, the improved plant GRAND pegRNA strategy from mammalian cells was successfully used to generate efficient insertion of protein tags in plants with a higher insertion efficiency of 25% [55]. Although all these studies promote its widespread application in plants, its low editing efficiency is still a concern and improved prime editors are needed to resolve these issues for a better use of this technology.

## 6. Application of CRISPR in Plant Abiotic and Biotic Stress Resistance

**Figure 4:**
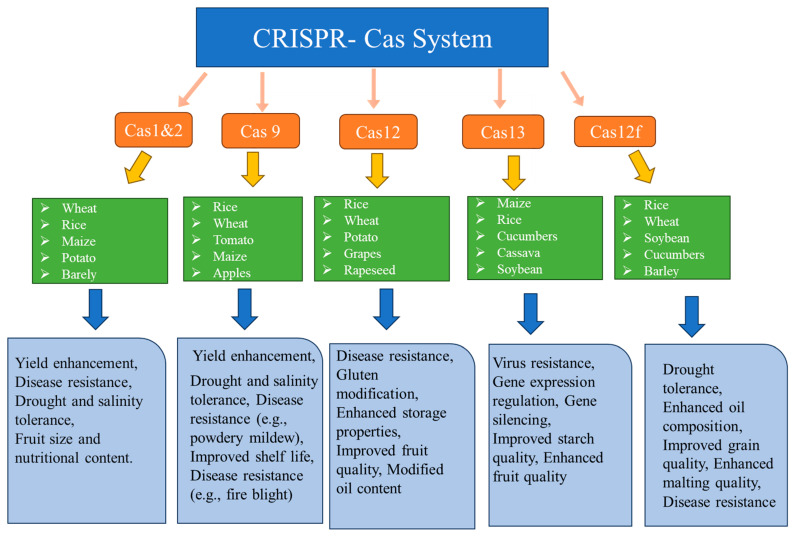
Use of CRISPR-Cas system in different crops.

CRISPR-Cas is a robust tool that can be used to knock-out, knock-in, or replace a gene element at a target genomic sequence to regulate the expression of a gene at the genome and epigenome level. This inexpensive and efficient tool has been employed to improve the traits of various crops (Figure 4).

### 6.1. Application of CRISPR on Plants

CRISPR-Cas9 genome editing can modify any gene in any type of plant. It enables faster genetic alteration than other methods due to its ease of use, effectiveness, affordability, and ability to target many genes. Plants that were previously disregarded can also be genetically modified using this technique. There is immense promise for both crop breeding and the advancement of sustainable agriculture [76]. CRISPR-Cas9 has produced impressive genetic modifications to improve metabolic pathways, resistance to biotic (fungal, bacterial, or viral pathogens) or abiotic (cold, drought, or salt) stresses, nutritional content, yield and grain quality, haploid seed production, herbicide resistance, and other traits. Significant examples include enhanced nutritional qualities in sorghum and wheat [78,79], resistance to diseases, and thermosensitive genic male sterility in maize [80] and wheat [81]. Potato plants that underwent one round of transfection through CRISPR-Cas9 technology were engineered to lack the gene responsible for making granule-bound starch synthase (GBSS). This modification led to the generation of potato crops capable of producing amylopectin starch, a trait highly valued in the commercial sector [82]. A cucumber CRISPR-Cas9 system was employed to deactivate the gene for the eukaryotic translation initiation factor, elF4E. This led to the creation of non-transgenic, identical mutant plants that were protected against the Cucumber vein yellowing virus (a type of Ipomovirus) and were also resistant to potyviruses, including Zucchini yellow mosaic virus and Papaya ring spot mosaic virus [83]. The potential to manage diseases for which no natural resistance has been found, like tomato brown rugose fruit virus and maize fatal necrosis disease, is enormous when genetic resistance to viruses and other pathogens is engineered [84]. Protoplasts of *N*. *tabaccum* are used by researchers to introduce donor DNA and CRISPR/Cas9 plasmids into regenerating plants. This tactic avoids stable nuclear transformation by depending on the temporary expression of the Cas9 protein [85]. In maize, a callus-specific CRISPR/Cas9 (CSC) system that uses the Cas9 gene driven by the promoters of ZmCTA1 and ZmPLTP enhances the production of heritable mutations while reducing somatic mutations. Crop genetic breeding can benefit greatly from the CRISPR/Cas9 system because of its precise editing capabilities [86].

### 6.2. Drawbacks of CRISPR-Cas for Plant Genome Editing

The process of using CRISPR/Cas genome editing (GE) is tough and demanding in trees with woody stems due to their extended growth periods, scarce availability of genetic mutations, and low success rate in genetic modification [87]. Therefore, depending on the goal of delivery, the adoption of an appropriate carrier may be taken into consideration in order to accomplish effective and quick delivery of the CRISPR/Cas system to plants. There are two types of delivery vectors: plasmid-based and viral/non-viral. The bean yellow dwarf virus, tobacco mosaic virus, potato virus X, and cowpea mosaic virus are among the viral vectors that have been employed in plants [88,89]. However, the use of big fragment sequences or even large Cas proteins is limited by the capacity of viral vectors, and the use of viral vectors may amplify the plant immune system’s defences. A range of materials, including inorganic nanoparticles, carbon nanotubes, liposomes, protein- and peptide-based nanoparticles, and nanoscale polymeric materials, are included in these non-viral vectors. When a target gene already exists and needs to be modified or rendered Inactive, CRISPR/Cas9-mediated genome editing can introduce minor substitutions or InDels at the target location. Foreign genes are inserted into plants and stably incorporated into the plant genome using Agrobacterium (Agrobacterium tumefaciens)-mediated transformation in the majority of plant genetic engineering cases. Because of constitutive gene expression, integrating the CRISPR/Cas9 cassette may result in undesired off-target consequences, plant mortality, and restrictions on carrying out functional research linked to particular developmental or physiological processes. Genetically modified organisms (GMOs) are a significant concern when foreign genes are present in chromosomes, even if they can be controlled through spatiotemporal gene expression with the use of recombinases and inducible promoters [90,91]. Actually, a few nations do not classify genome-edited crops as genetically modified organisms (GMOs), so they can be grown without the usual limitations that come with them [92]. Because of this, the primary method for producing transgene-free genome-edited plants is time-consuming and difficult genetic segregation, which can be particularly difficult for crops with large polyploid genomes. As a result, numerous methods are being created and put into use to more accurately identify transgene-free plants.

### 6.3. CRISPR in Abiotic Stress Resistance

Abiotic stresses like drought, salinity, heavy metals, and extreme temperatures greatly affect plant growth and development and could lead to 50% of crop losses [93]. According to a report, 20% of the arable land is affected by salinization, and an increasing percentage of Earth’s land is expected to be affected in coming years at a faster pace [94]. Plants under salt stress may have negative effects that lower yield and quality. This is due to the induction of osmotic, ionic, and secondary stress. Using the CRISPR-Cas9 system, Alam et al. in 2022 [78,95] developed a rice variety by knocking out the OsbHLH024 gene and enhancing the expression of the ion transporter genes OsHKT1-3, OsHAK7, and OsSOS1 [96]. CRISPR/Cas9-induced OsRR22 gene mutation in rice increased its resistance to salt without affecting other agronomic characteristics [97]. It was also employed successfully to alter OsRAV2, and the resultant mutant exhibited increased survivability under salt stress. Furthermore, it has been reported that the CRISPR-Cas9 system can significantly increase the resistance of different crops to salt stress by deleting or overexpressing the genes, for example, tomato SlARF4, rice OsDST [98], OsNAC041 and OsmiR535 [99], and barley HvITPK1 [9], Arabidopsis AtC/VIF1 [100], and Soybena GmMYB118 transcription factor [101]. The increasing impact of global warming has led to increased drought and temperature stress conditions, and by targeting the drought response genes using the CRISPR-Cas9 system, enhanced drought tolerance has been achieved in various plant species. In rice, improved drought tolerance was achieved by targeted mutagenesis of OsERA1 using CRISPR/Cas9 [102]. Other genes of rice, viz. OsDST, SRL1, SRL2, and SAPK2, have been edited to improve the plant’s ability to have better water retention capacity, lower stomatal density, improved scavenging of reactive oxygen species, and have improved drought tolerance [98,103,104]. The ospyl9 mutant, produced using CRISPR/Cas9, was shown to boost up rice yield and drought tolerance [73]. Additional studies involving CRISPR/Cas editing of ERF family members from rice (OsBIERF1, OsBIERF3, and OsBIERF4), maize (ARGOS8), and wheat (TaERF3) have also been shown to improve drought tolerance [104,105]. Ding et al., 2020 suggest that cold stress, encompassing temperatures lower than 20 °C and 0 °C, obstructs growth and plant development and significantly restricts plant geographical distribution and agricultural output [106]. Low temperatures directly decrease plants’ metabolic response, causing osmotic stress, oxidative stress, and other types of stress. CRISPR/Cas9 editing was used to create rice with pyl1/4/6 triple knockdown. The mutant showed less germination before harvest, a higher yield, and a higher tolerance to temperature than the natural variety. Genes involved in ABA signalling and cell membrane biosynthesis have been targeted in rice using the CRISPR-Cas9 system (CBF1, OsANN3, SAPK2, and OsMYB30) and have been found to prevent electrolyte leakage, improve relative electrical conductivity and improve drought tolerance efficiency by nearly 63% [104,107]. Other major abiotic stresses like high temperature and heavy metal stress have also been addressed successfully using CRISPR gene editing in various crops like tomato (BZR1 and AGL6 for heat stress tolerance), maize (TGMS5 for heat tolerance) and rice (OsNRAMP1 and OsNRAMP5 for cadmium tolerance, OsARM1 for arsenic tolerance, and OsHAK1 for caesium tolerance) [105,108]. The list of crops edited using the CRISPR-Cas system for improvement of abiotic stress tolerance has been summarized in (Table 6).

### 6.4. CRISPR in Biotic Stress Resistance

Crop yield losses up to 20–40% worldwide are accounted to various biotic factors like viruses, fungi, and bacteria [110]. The CRISPR-Cas system has proven as a robust tool that could specifically knockout undesirable genes and confer plant tolerance to various diseases. Powdery mildew is one of the most destructive types of fungal diseases that crops can suffer, as it significantly reduces crop yield. Higher resilience to powdery mildew in wheat was achieved by knocking out three MILDEW-RESISTANCE LOCUS (MLO) genes, which are known to be responsible for the infection, using CRISPR/Cas9. Similarly, in grapes [111] and tomatoes [112], resistance to powdery mildew was achieved through CRISPR/Cas9-mediated reduction in SlMLO and VvMLO. Furthermore, tomato powdery mildew resistance was markedly enhanced by the CRISPR/Cas9-mediated SlPMR4 mutation, although immunity was not entirely restored [113]. Several other devastating fungal diseases like Fusarium wilt and blast cause severe crop losses, and the lack of any resistant germplasm restricts the possibility of traditional breeding for developing resistant varieties [114]. The CRISPR-Cas system was successfully used to edit genes and confer resistance to these pathogens. In rice, OsERF922 and OsSEC3A genes were mutated using CRISPR/Cas9 and the plants tested were shown to be significantly resistant to blast disease at both the seedling and tillering stages [25,109]. A method enabled by CRISPR/Cas9 technology allows for the mutation of the acetylegenase-encoding genes ACER1a and ACET1b to generate distinct resistant materials with increased resistance to bacterial and fungal diseases. Tomato susceptibility gene SlDMR6-1 mutations produced by CRISPR/Cas9 offer resistance against a variety of diseases, such as bacteria, oomycetes, and fungus [115]. Broad-spectrum resistance to bacteria and fungi was demonstrated by CRISPR/Cas9-mediated osnramp1 mutants, which showed decreased catalase (CAT) activity but elevated hydrogen peroxide (H_2_O_2_) content and superoxide dismutase (SOD) activity [116]. The list of crops and the genes edited in different crops for abiotic stress tolerance are summarized in (Table 7 and Table 8).

### 6.5. Application of CRISPR on Animals

In scientific research, animal models are crucial for the study of disease mechanisms, the creation of new medications, and the production of agricultural goods [126]. Researchers frequently genetically alter animals to produce desired features in order to build optimal animal models. According to Ribitsch et al. (2020), gene-modified small rodent models, particularly mice and rats, offer a wealth of experimental data and are crucial for studying both important biological functions and disease mechanisms [127]. However, these mini-animal prototypes also possess a few limitations. Firstly, due to the significant physiological, anatomical, and genomic differences between humans and small animals, it is frequently impossible for researchers to fully understand the pathogenesis of diseases using small-animal models to replicate the symptoms of human diseases. This has also resulted in the clinical trial failure of numerous medications that were evaluated using small-animal models [26,128]. Furthermore, small-animal models are used less frequently in agricultural processes like producing animal byproducts. Researchers are more frequently employing big-animal models, like non-human primates (NHPs), pigs, dogs, goats, and sheep, to investigate human illnesses because of their resemblances in genetics, physiology, developmental biology, social behaviour, and intelligence. However, the development of large-animal models with altered genes has been hampered by challenges with genome editing. Animal-model gene editing has become much more efficient thanks to advances in gene editing technology like CRISPR/Cas9, zinc finger nucleases (ZFNs), and transcription activator-like effector nucleases (TALENs). ZFNs and TALENs have been applied to a number of species; however, because large and small animals differ, it is still difficult to create large-animal models with ZFNs. The most widely used and successful gene editing technique is CRISPR/Cas9, which combines cleavage and recognition elements like Cas9 nuclease and single-guide RNAs. The method has been quickly optimised by researchers, who have then used it to modify the genes of small animals including mice, rats, and zebrafish.

## 7. Crop Improvement, Possible Risks, and Ethical Concerns of CRISPR-Cas-Based Genome Editing

The applications of genetic modification technologies based on CRISPR are applicable in many domains, such as agricultural enhancement and plant functional genomics [9]. Because of its ease of application, CRISPCas9 technology is easily accessible and relatively inexpensive to use. Public sector institutions, including the Consultative Group for International Agricultural Research (CGIAR) are using this technology to help smallholder farmers to improve crops of less importance to the profit-driven private sectors, and the current genome editing projects conducted by CGIAR to improve several crop traits are detailed by Pixley et al., 2022 [129]. Genome editing is being applied to more than 40 crops to improve food and feed quality or stress tolerance in more than 25 countries, but only six genome-edited crops for different traits have been approved for commercialization to date [130]. Although the CRISPR-Cas system has great potential for use, many countries are unsure about the regulations to be used for growing these genome-edited crops [131]. Other major risk factors include generation of off-target mutations [117] and possible break down of natural reproductive barriers that prevent some mutations from occurring in nature [75]. However, much effort is being made to improve computational and bioinformatic tools to minimize off-target edits and to better understand the nature and frequency of these non-target mutations generated using CRISPR technology. Moreover, it is also important to consider that natural mutations occur during every generational advance during traditional plant breeding, and the frequency of mutations generated through chemicals or radiation is 1000-times higher than the natural mutation frequency and the frequency of the non-target mutation generated by current genome editing tools [75,132]. Hence, it is important to assess the risk of using the CRISPR-based technology over other traditional methods of plant breeding.

The remarkable potential that CRISPR-Cas9 technology unlocks also raises important ethical and regulatory issues. The act of modifying the genetic code of live beings gives rise to important inquiries on the limits of genetic engineering and the possible consequences of modifying inherent biological functions. The moral implications of using CRISPR-Cas9 for germline editing, in which genetic modifications that are inherited by offspring can be passed down to them, have long been a topic of discussion. The complexities of germline modifications require careful thought in order to avoid unexpected outcomes and to guarantee the highest level of responsibility in its implementation. Moreover, the regulatory environment surrounding CRISPR-Cas9 technology is constantly changing, with various nations and authorities taking distinct stances on its supervision. It becomes essential to establish clear rules and regulations governing the use of CRISPR-Cas9 in order to ensure its ethical use, responsible execution, and steadfast dedication to environmental and human safety.

## 8. Conclusions and Perspectives

Plant gene editing can be accomplished with ease using the easy-to-use, accurate, and user-friendly CRISPR-Cas toolkit. CRISPR is a technique that is utilised for many different purposes, such as improving global food security, introducing foreign genes in synthetic biology, and clarifying the structure and function of plant genomes. Additionally, it can be applied to both domesticated and non-domesticated plants at several loci to enhance specificity and efficiency through multiple gene editing. Gene drives can eradicate weeds and pests, but until a robust, regulated framework is available, regulatory bodies and researcher societies should work together to prevent illicit genome editing. Nowadays, CRISPR/Cas9 technology is being used to improve different traits such as abiotic stress tolerance, disease resistance, quality, and yield of both monocots and dicots. Crop genomic sequences can be modified to provide higher yields by diversifying the approaches used to characterise the activities of individual genes. The Cas proteins have boosted basic, therapeutic, and diagnostic research. Because these CRISPR/Cas systems are inexpensive and simple to use, many researchers are using them to investigate the functions of different organisms’ genes. Furthermore, identifying the unexplained evolution of Cas masteries, which persist in a variety of microscopic organisms or archaea, would transform a variety of fields, such as the diagnosis of new illnesses, medicines, agriculture, breeding, and so forth. If these proteins’ advanced genome-editing potential is fully explored, it may ignite an untapped CRISPR “fever” that might lead to the widespread adoption of compelling and revolutionary CRISPR technologies in the near future.

## Figures and Tables

**Table 1:** Classification of CRISPR-Cas system: Types of Cas proteins with their target molecule.

CRISPR/Cas System Used	Host Organism	Name of the Effector	Types of Protein	Target Molecule	Reference
Cas7, Cas5, Cas8, and Cas3	*Escherichia coli*	Cas3, Cascade, and crRNA	Cas 3	ssDNA	[12]
Cas7, Cas5, and Cas1	*Staphylococcus epidermidis*	Cmr/Csm, crRNA, and Cas10	Cas 6	ssDNA	[13]
Cas7, Cas5, and Csf1	*-*	-	Csf1	-	[14]
Cas9	*Streptococcus thermophilus* and *Streptococcus pyogenes*	Cas9, tracrRNA, and crRNA	Cas9	dsDNA	[15]
Cas12	*Francisella novicida*	Cpf1, crRNA, and tracrRNA	Cpf1	ssDNA and ds DNA	[16]
Cas13	-	C2c1 and crRNA	C2c2	ssRNA	[17]

**Table 2:** Host organisms with their Cas protein and target molecules.

Host Organism	Target Molecule	Protein Name	References
*Campylobacter jejuni*	DNA	Cas9	[19]
*Streptococcus thermophilus*	DNA	Cas9	[20]
*S. thermophilus*	DNA	Cas9	-
*Neisseria meningitidis*	DNA	Cas9	[21]
*Staphylococcus aureus*	DNA	Cas9	[20]
*F. novicida*	DNA	Cas9	-
*S. pyogenes*	DNA	Cas9	[22]
*S. pyogenes*	dsDNA	Cas9	[23]
*Acidaminococcus species*	DNA	Cpf1	[24]
*Parafrancisella*	DNA	Cpf1	[25]
*Alicyclobacillus acidoterrestris*	DNA	C2c1	[26]
*Acidaminococcus species*	DNA	Cas12a	[19]
*Lactobacillus acidophilus*	ssRNA	Cas13	[17]
*Uarchaea*	ssDNA	Cas12f	[27]
*A. species*	dsDNA	Cas12i	[28]
*Nicotiana benthamiana*	DNA	Cas12j	[29]

**Table 3:** Major naturally occurring and genetically modified Cas enzymes used for genome editing.

Name	Cas	CRISPR/Cas	PAM	PAM Location	Resources	Reference
SpRY	Cas9	Type II	NRN or NYN	3′	Engineered SpCas9	[30]
SpG	Cas9	Type II	NGN	3′	Engineered SpCas9	[30]
Cas9-NRNH	Cas9	Type II	NRNH	3′	Engineered SpCas9	[31]
HypaCas9	Cas9	Type II	NGG	3′	Mutated SpCas9-HF	[32]
evoCas9	Cas9	Type II	NGG	3′	Mutated SpCas9	[33]
Sniper-Cas9	Cas9	Type II	NGG	3′	Engineered SpCas9	[34]
xCas9	Cas9	Type II	NG	3′	Engineered SpCas9	[35]
SpCas9-NG	Cas9	Type II	NG	3′	Engineered SpCas9	[36]
eSpCas9	Cas9	Type II	NGG	3′	Engineered SpCas9	[37]
SpCas9-HF	Cas9	Type II	NGG	3′	Engineered SpCas9	[38]
SaCas9-KKH	Cas9	Type II	NNNRRT	3′	Engineered SaCas9	[39]
Modified SpCas9	Cas9	Type II	NGA or NAG	3′	Engineered SpCas9	[40]
FnCas9variant	Cas9	Type II	YG	3′	Modified FnCas9	[41]
SpCas9	Cas9	Type II	NGG	3′	*S. pyogenes*	[15]
SaCas9	Cas9	Type II	NNGRRT	3′	*S. aureus*	[41]
FnCas9	Cas9	Type II	NGG	3′	*F. Novicida*	[40]
NmCas9	Cas9	Type II	NNNNGATT	3′	*N. meningitidis*	[42]
CjCas9	Cas9	Type II	NNNNRYAC	3′	*C. jejuni*	[19]
St1Cas9	Cas9	Type II	NNAGAAW	3′	*S. thermophilus*	[43]
St1Cas9	Cas9	Type II	NGGNG	3′	*S. thermophilus*	[43]
FnCas12a	Cas12a(cpf1)	Type II	TTTN or YTN	5′	*F. novicida*	[23]
LbCas12a	Cas12a(cpf1)	Type II	TTTV	5′	*L. bacterium*	[23]
AsCas12a	Cas12a(cpf1)	Type II	TTTV	5′	*Acidaminococcus* sp.	[44]
LsCas13#	Cas13(C2c2)	TypeVI	NA	NA	*L. shahii*	[17]
Cas14	Cas14	NA	NA	NA	*Archaea*	[27]

**Table 4:** Comparison of Cas9 vs Cas12 vs Cas13 vs Cas14.

Parameter	Cas9	Cas12	Cas13	Cas14
Size of Protein (Amino Acid)	~1000–1600	~1300	~1400	~400–700
Target	DNA	DNA	RNA	DNA
RNA	Two RNA molecules	Single RNA molecules	Two RNA molecules	Single RNA molecules
Nuclease Site	2 nuclease domains HNH and RuvC	Single-nuclease RuvC-Nuc	Target-RNA domain HEPN	DNA-binding domain RuvC
Pattern of cut	Blunt	Sticky-ended	Degraded	NA
Spacer Size	16–20 nt	16–25 nt	25–35 nt	NA
Protospacer restriction	PAM	PAM	PFS	PAM
Single guide molecular size (Nucleotides, nt)	17–24 nt	42–44 nt	−64 nt	−140 nt
Non-specifically cut nucleic acids (DNA or RNA)	DNA (SS)	DNA (SS)	RNA (SS)	DNA (SS)

**Table 5:** Mechanism and its merits of Cas protein.

Cas Proteins	Mechanism	Applications	Merits	Demerits	Reference
Cas 9	Guide RNA Recognition DNA Binding DNA Cleavage	Gene Editing Gene Regulation Functional Genomics	Target-Specific Editing	Off-Target Effects	[16]
Cas 12	Guide RNA Recognition Target Binding DNA Cleavage	Gene Editing	The Capacity to Accurately Target Desired Sequences	Off-Target Effects	[67]
Cas 13	Guide RNA Recognition RNA Binding RNA Cleavage	RNA Interference RNA Editing	RNA Targeting	Off-Target Effects	[17]
Cas 12f	Guide RNA Recognition DNA Binding DNA Cleavage	Gene Editing	Target Specificity	Off-Target Effects	[68]

**Table 6:** CRISPR/Cas genes reported to confer tolerance against different abiotic stresses.

Stress	Cas Enzmyes	Crop	The Name of the Target Gene	References
Salinity	Cas9	Rice (*Oryza sativa*)	BASIC HELIX-LOOP-HELIX 024 (OsbHLH024)	[95]
		Rice (*Oryza sativa*)	RESPONSE REGULATORS 22 (OsRR22)	[97]
		Rice (*Oryza sativa*)	RELATED TO ABI3/VP1 2 (OsRAV2)	[73]
		Rice (*Oryza sativa*)	DROUGHT AND SALT TOLERANCE (OsDST)	[73]
		Rice (*Oryza sativa*)	NAM, ATAF, and CUC 041 (OsNAC041)	[109]
		Rice (*Oryza sativa*)	OsmiR535	[99]
		Barley (*Hordeum vulgare*)	INOSITOLTRISPHOSPHATE 5/6 KINASES 1 (HvITPK1)	[9]
		Tomato (*Solanum lycopersicum*)	HYBRID PROLINE-RICH PROTEIN 1 (SlHyPRP1)	[73]
		Tomato (*Solanum lycopersicum*)	Auxin Response Factor 4 (SlARF4)	[73]
Drought		Rice (*Oryza sativa*)	ENHANCED RESPONSE TO ABA1 (OsERA1)	[102]
		Rice (*Oryza sativa*)	OsDST	[98]
		Rice (*Oryza sativa*)	PYRABACTIN RESISTANCE-LIKE 9 (OsPYL9)	[99]
		Rice (*Oryza sativa*)	SEMI-ROLLED LEAF 1 (SRL1) and SEMI-ROLLED LEAF 2 (SRL2)	[9]
		Maize (*Zea mays*)	AUXIN-REGULATED GENE INVOLVED IN ORGAN SIZE 8 (ZmARGOS8)	[73]
	Cas9	Wheat (*Triticum aestivum*)	DEHYDRATION RESPONSIVE ELEMENT BINDING PROTEIN 2 (TaDREB2)	[19]
		Wheat (*Triticum aestivum*)	ETHYLENE-RESPONSE FACTOR 3 (TaERF3)	[19]
		Tomato (*Solanum lycopersicum*)	GA-INSENSITIVE DWARF1 1 (SlGID1)	[73]
		Tomato (*Solanum lycopersicum*)	LATERAL ORGAN BOUNDARIES DOMAIN 40 (SlLBD40)	[46]
Arsenic Caesium	Cas 9	Rice (*Oryza sativa*)	HIGH-AFFINITY POTASSIUM TRANSPORTER 1 (OSHAK1)	[73]
		Rice (*Oryza sativa*)	ARSENITE-RESPONSIVE MYB1 (OsARM1)	[73]
Low temperature		Rice (*Oryza sativa*)	PIN-FORMED 5b (OsPIN5b)	[73]
	Cas9	Rice (*Oryza sativa*)	GRAIN SIZE (GS3)	[73]
		Rice (*Oryza sativa*)	V-MYB AVIAN MYELOBLASTOSIS VIRAL ONCOGENE HOMOLOG 30 (OsMYB30)	[73]
High temperature		Rice (*Oryza sativa*)	PYRABACTIN RESISTANCE-LIKE 1/4/6 (OsPYL1/4/6)	[73]
	Cas9	Tomato (*Solanum lycopersicum*)	MITOGEN-ACTIVATED PROTEIN KINASES 3 (SlMAPK3)	[73]
Cadmium		Rice (*Oryza sativa*)	NATURAL RESISTANCE-ASSOCIATED MACROPHAGE PROTEIN 5 (OsNRAMP5)	[26]
	Cas9	Rice (*Oryza sativa*)	LOW-AFFINITY CATION TRANSPORTER 1 (OsLCT1)	[73]
		Rice (*Oryza sativa*)	NATURAL RESISTANCE-ASSOCIATED MACROPHAGE PROTEIN 1 (OsNRAMP1)	[26]

**Table 7:** Achievement of different targeted gene.

Crop Species	Trait	Gene Targeted	Achievement Made	Reference
*Oryza sativa*	Disease resistance Thermotolerance Grain-length salt tolerance	Mutation and loss of function in OsSWEET14 Mutation in EBEs of OsSWEET14 gene Mutations in EBE of three promoters of SWEET11, SWEET13, and SWEET14 Knockdown of the Os8N3 in rice	Mutant rice confers strong resistance to African Xoo and Asian Xoo strains Enhanced resistance to locally isolated virulent Xoo strains. Stable transgenic rice lines indicated robust, broad-spectrum resistance to Xoo. Transmission of mutations to future generations, and enhanced resistance to Xoo in homozygous mutants.	[117]
*Musa balbisiana*	Enhanced resistance	Mutation in downy mildew resistance 6 (DMR6)	Musa dmr6 transgenic mutants of banana showed enhanced resistance to BXW, and did not show any detrimental effect on plant growth.	[12]
*Musca domestica*	Disease resistance	Mutation in apple DIPM-1, DIPM-2, and DIPM-4	Resistance to fire blast disease in non-transgenic but mutant apple lines.	[13]
*Solanum lycopersicum*	Disease resistance	Loss of function mutation in SlDMR6-1 gene	Mutants do not have detrimental effects on growth and had multiple disease resistance to *P. syringae*, *P. capsici*, and *Xanthomonas* spp.	[14]
*Citrus sinensis Osbeck*	Canker resistance	Mutation and loss of function in CsWRKY22	Mutant orange plants showed decreased susceptibility to citrus canker.	[15]
*C. sinensis Osbeck*	Canker resistance	CRISPR/Cas9-targeted mutation in CsLOB1 promoter in citrus	Promoter editing of CsLOB1 alone was sufficient to enhance citrus canker resistance in citrus.	[17]

**Table 8:** CRISPR/Cas genes intended to confer tolerance against biotic stress.

Stress	Pathogen Factor	Crop	The Name of the Target Gene	References
Insect disease	Plant hopper	Rice (*Oryza sativa*)	CYTOCHROME P450 71A1 (OsCYP71A1)	[68]
	Stem borer	Rice (*Oryza sativa*)	OsCYP71A1	[68]
	Common cutworm	Soybean (*Glycine max*)	CALCIUM-DEPENDENT PROTEIN KINASE 38 (GmCDPK38)	[118]
Virus disease	Rice tungro spherical virus	Rice (*Oryza sativa*)	eIF4G	[119]
	Cucumber vein yellowing virus	Cucumber (*Cucumis sativus*)	EUKARYOTIC TRANSLATION INITIATION FACTOR 4E (eIF4E)	[83]
	Zucchini yellow mosaic (Cucumis virus)	Cucumber (*Cucumis sativus*)	eIF4E	[83]
	Papaya ring spot mosaic (Cucumis virus)	Cucumber (*Cucumis sativus*)	eIF4E	[83]
	Tomato mosaic virus	Tomato (*Solanum* *lycopersicum*)	DICER-LIKE 2b (SlDCL2b)	[111]
	Potato virus X	Tomato (*Solanum lycopersicum*)	SlDCL2a and SlDCL2b	[111]
Fungus disease	Rice Blast	Rice (*Oryza sativa*)	OsERF922 SUBUNIT OF THE	[111]
		Rice (*Oryza sativa*)	EXOCYST COMPLEX 3A (OsSEC3A)	[109]
		Rice (*Oryza sativa*)	Pi21 and Bsr-d1	[25,96]
	Powdery mildew	Tomato (*Solanum lycopersicum*)	MILDEW RESISTANT LOCUS O (SlMLO)	[120]
		Wheat (*Triticum aestivum*)	TaMLO-A1, TaMLO-B1 and TaMLO-D1	[121]
		Grapevine (*Vitis vinifera)*	VvMOL3	[121]
		Tomato (*Solanum lycopersicum*)	POWDERY MILDEW RESISTANCE 4 (SlPMR4)	[112]
	Late blight	Tomato (*Solanum lycopersicum*)	miR482b and miR482c	[111]
	Gray mould	Tomato (*Solanum lycopersicum*)	PECTATE LYASE (SlPL)	[122]
Bacterial disease	Bacterial blight	Rice (*Oryza sativa*)	SUGARS WILL EVENTUALLY BE EXPORTED TRANSPORTER 13 (OsSWEET13)	[123]
	Citrus bacterial canker	Orange (*Citrus sinensis*)	LATERAL ORGAN BOUNDARY 1 (CsLOB1)	[124]
	Bacterial leaf spot disease	Tomato (*Solanum lycopersicum*)	JASMONATE ZIM-DOMAIN 2 (SlJAZ2)	[125]

## Data Availability

The findings of this study are available from the corresponding author.
